# Glutaminyl cyclase-mediated toxicity of pyroglutamate-beta amyloid induces striatal neurodegeneration

**DOI:** 10.1186/1471-2202-14-108

**Published:** 2013-10-01

**Authors:** Andreas Becker, Stephanie Kohlmann, Anca Alexandru, Wolfgang Jagla, Fabio Canneva, Christoph Bäuscher, Holger Cynis, Reinhard Sedlmeier, Sigrid Graubner, Stephan Schilling, Hans-Ulrich Demuth, Stephan von Hörsten

**Affiliations:** 1 Department of Experimental Therapy, Friedrich-Alexander-University Erlangen-Nürnberg, Palmsanlage 5, 91054 Erlangen, Germany; 2 Ingenium Pharmaceuticals GmbH, 82152 Martinsried, Germany; 3 Probiodrug AG, Biocenter, Weinbergweg 22, 06120 Halle (Saale), Germany

**Keywords:** ETNA, Pyroglutamate Aβ, Glutaminyl cyclase, Alzheimer’s disease, TBA, Neurodegeneration, Neuroinflammation, Striatum

## Abstract

**Background:**

Posttranslational modifications of beta amyloid (Aβ) have been shown to affect its biophysical and neurophysiological properties. One of these modifications is N-terminal pyroglutamate (pE) formation. Enzymatic glutaminyl cyclase (QC) activity catalyzes cyclization of truncated Aβ(3-x), generating pE3-Aβ. Compared to unmodified Aβ, pE3-Aβ is more hydrophobic and neurotoxic. In addition, it accelerates aggregation of other Aβ species. To directly investigate pE3-Aβ formation and toxicity *in vivo,* transgenic (tg) ETNA (E at the truncated N-terminus of Aβ) mice expressing truncated human Aβ(3–42) were generated and comprehensively characterized. To further investigate the role of QC in pE3-Aβ formation *in vivo*, ETNA mice were intercrossed with tg mice overexpressing human QC (hQC) to generate double tg ETNA-hQC mice.

**Results:**

Expression of truncated Aβ(3–42) was detected mainly in the lateral striatum of ETNA mice, leading to progressive accumulation of pE3-Aβ. This ultimately resulted in astrocytosis, loss of DARPP-32 immunoreactivity, and neuronal loss at the sites of pE3-Aβ formation. Neuropathology in ETNA mice was associated with behavioral alterations. In particular, hyperactivity and impaired acoustic sensorimotor gating were detected. Double tg ETNA-hQC mice showed similar Aβ levels and expression sites, while pE3-Aβ were significantly increased, entailing increased astrocytosis and neuronal loss.

**Conclusions:**

ETNA and ETNA-hQC mice represent novel mouse models for QC-mediated toxicity of truncated and pE-modified Aβ. Due to their significant striatal neurodegeneration these mice can also be used for analysis of striatal regulation of basal locomotor activity and sensorimotor gating, and possibly for DARPP-32-dependent neurophysiology and neuropathology. The spatio-temporal correlation of pE3-Aβ and neuropathology strongly argues for an important role of this Aβ species in neurodegenerative processes in these models.

## Background

The formation of pyroglutamate (pGlu, pE) at the N-terminus of various peptides and proteins is catalyzed by glutaminyl cyclase (QC, QPCT) and its isoenzyme (isoQC, QPCTL). Alternatively, glutamine (Q) tends to form pGlu spontaneously under mild conditions at significant rates, which contrasts with glutamate (E) [1-3]. Likewise, glutaminyl peptides are preferred substrates for both QC and isoQC.

Pyroglutamate decreases solubility of beta amyloid (Aβ), prevents further enzymatic degradation, and increases stability [4,5] which may intensify the toxicity of several key proteins in a number of neurodegenerative diseases.

At present there are several hypotheses for posttranslationally modified proteins accounting for neuropathology in Parkinson’s disease and Huntington’s disease (HD) [6] as well as experimental evidence in familial British and Danish dementia [7-10] and Alzheimer’s disease (AD) [11].

In case of AD, Aβ is generated in the amyloidogenic pathway, a multi-step cleavage process of the amyloid precursor protein (APP) [12,13] and pyroglutamate Aβ (pE3-Aβ) is formed enzymatically during posttranslational peptide maturation via cyclization of N-terminal glutamate residues of truncated Aβ(3-×) species by QC-like enzymatic activity [2,14]. The characteristic properties of pE3-Aβ, high aggregation propensity and stability, as well as a strong tendency to seed further aggregation of other Aβ species [9,15,16], result in an Aβ form with increased toxicity.

Recently, QC-dependent formation of small amounts of pE3-Aβ inducing decreased synaptic plasticity, progressive neuronal loss and astrocytosis resulting in an early neurodegenerative phenotype, has been demonstrated [17].

The TBA2.1 mouse line described by Alexandru et al. and other lines, namely TBA2 [18] and FAD42 [19] use truncated Aβ constructs with an E to Q substitution at position 3 of the Aβ peptide. The N-terminal Q residue of the used Aβ(Q3-42) construct is the preferred substrate for QC, but it shows a higher rate of spontaneous cyclization, compared to the endogenous Aβ species with E at position 3 [2,3].

In line with this research, several new transgenic (tg) ETNA (E at the truncated N-terminus of Aβ) mouse models were generated using a truncated, but non-mutated and therefore more physiological Aβ(E3-42) construct to analyze endogenous Aβ(pE3-42) formation *in vivo*.

ETNA mice were intercrossed with tg mice overexpressing human QC (hQC) to further investigate the role of QC in pE3-Aβ formation *in vivo*. Interestingly, these newly generated mouse lines show significant neuronal loss in striatum, substantiating the hypothesis of modified Aβ as crucial molecular player in neurodegeneration.

## Methods

### Experimental animals

Tg ETNA mice were generated by chromosomal integration of an expression cassette as described for TBA2.1 and TBA2.2 [17]. In brief, murine Thy1.2 regulatory sequences flank the coding sequence for a fusion protein consisting of the pre-pro peptide of murine thyrotropin-releasing hormone (TRH, Thyroliberin) fused to the N-terminus of the truncated human Aβ(3–42) and directs expression into neuronal tissue [20]. The regulatory elements prohormone convertase (PC) cleavage within the trans-Golgi and secretory vesicles liberates Aβ(3–42) preferentially within the secretory pathway, where they are modified by QC activity [21,22].

Similarly, tg hQC mice were generated by integration of a Thy1.2 expression cassette containing the coding sequence of hQC (NM_012413) into the chromosome of CBA/C57BL/6 hybrid embryos. F1 tg animals which yielded strong genomic southern blot hybridization signals, using labeled expression cassette probes, were selected for colony expansion and backcrossed to C57BL/6 wildtype (wt) animals. Transgene expression levels in the established colonies were characterized by real-time quantitative reverse transcription PCR (qRT-PCR). The mouse lines E5, E8, and hQC displayed high transgene transcription rates and were selected for phenotypic analysis.

The following genotypes of tg and control animals were used (Table 1). Heterozygous (het) and homozygous (hom) E5 were compared to wt littermates. E8 het and E8 hom were compared to wt littermates. By intercrossing E5 and E8, double heterozygous E85 het/het were generated and compared to wt/wt littermates.

**Table 1:** Genotypes of transgenic animals

**ETNA nomenclature**		**E8**	**E5**	**hQC**
E5	wt	-	wt	-
E5	het	-	het	-
E5	hom	-	hom	-
E8	wt	wt	-	-
E8	het	het	-	-
E8	hom	hom	-	-
E85	wt/wt	wt	wt	-
E85	het/het	het	het	-
E8-hQC	wt/wt	wt	-	wt
E8-hQC	hom/wt	hom	-	wt
E8-hQC	hom/het	hom	-	het
E8-hQC	hom/hom	hom	-	hom
E85-hQC	het/het/wt	het	het	wt
E85-hQC	het/het/hom	het	het	hom

Overview of used genotypes and nomenclature of ETNA models. Wildtype (wt), heterozygous (het), and homozygous (hom) animals of single transgenic E5 and E8 models were used.

For the double transgenic E85 model, the E8 and E5 models were crossbred and only double heterozygous (het/het) and wildtype (wt/wt) animals were used.

E8 and E85 animals were intercrossed with hQC animals to generate multi-tg ETNA-hQC animals. In these models the genotype of the hQC allele is always indicated last.

For the double transgenic E8-hQC model, In E8-hQC the E8 allele was always homozygous in hom/wt, hom/het, and hom/hom animals and wildtype (wt/wt) animals were used as control.

For the triple transgenic E85-hQC model the E8 and E5 alleles were always double heterozygous (het/het) and wt, and hom of the hQC allele was used.

E8 and E85 were intercrossed with hQC to generate multi-tg E8-hQC and E85-hQC. In the genotype description of multi-tg ETNA-hQC animals, the hQC allele is always indicated last. E8-hQC hom/wt, hom/het, and hom/hom were compared to wt/wt littermates. E85-hQC always refers to het/het for the E85 alleles and both wt and hom of the hQC allele were used. As no gender differences were observed in these lines, both male and female animals were used in this study. For all experiments, mice of the age 1 to 14 months were used, while the maximum age difference in one group was 7 days.

### Animal care

Laboratory animal care and experiments were conducted in accordance with the German animal protection act and the regulations of the government of Upper Bavaria (Regierung Oberbayern; permission TVA 55.2-1-54-2531-135-07). In this proposal the role of the enzyme QC in the development of AD should be investigated. Thereby the question should be addressed, if a genetic modulation of Aβ or a pharmacological inhibition of QC-activity can achieve a causal therapy of AD. Animal protocols were designed to minimize any pain and stress experienced by the animals. Prior to the study, the number of required animals was statistically determined and not exceeded during the experiments. Number of animals and all animal protocols were specifically approved by the governmental animal ethics committee of Upper Bavaria. Health and immune status of all experimental animals was constantly monitored.

### Animal housing and husbandry

All mice were maintained in individually ventilated cages (IVC; Tecniplast, cage Euro-standard Type II L) animal units in a temperature (22 +/−2°C) and humidity (55 +/− 10%) controlled facility with a 12 h light/dark cycle (lights on at 6 a.m.), using standard sterile bedding and had access to standard laboratory pellets (ssniff Spzialdiät) and water *ad libitum*.

### Preparation of brain tissue

Mice were transferred to the preparation laboratory, deeply anesthetized with carbon dioxide, and transcardially perfused with phosphate-buffered saline (PBS). Brains were removed from the skull and sagitally bisected. Both hemispheres were used for different analyses to reduce the number of used animals and to correlate neurodegeneration and protein levels per animal. Left hemispheres were snap-frozen in liquid nitrogen and stored at −80°C for analysis of enzyme activity, mRNA or protein levels. Right hemispheres were cut into sample pieces and immersion-fixed in IHC Zinc Fixative (BD Pharmingen) according to the manufacturer’s protocol.

### Genetic characterization of transgenic models

Mapping of the chromosomal transgene integration sites for ETNA and hQC lines was performed as described before [23] using the Genome Walker Universal Kit (Clontech). ETNA transgene expression levels were assessed by qRT-PCR. In brief, brain biopsies were homogenized in QIAzol reagent (Qiagen) using an Ultra Turrax disperser (Sigma) and, after centrifugation, total RNA was further purified from the aqueous phase using RNeasy spin columns (Qiagen). 1 μg RNA was reverse transcribed using oligodT primers and Superscript II (Invitrogen) according to manufacturer’s instructions. PCR reactions were performed in duplicates using 1 μl of resulting cDNA per 20 μl reaction volume containing QuantiTect SYBR Green PCR Master Mix (Qiagen). The housekeeping gene beta-actin (ACTB) was used as control. Two ETNA transgene-specific primer sets were used for PCR amplification with one set containing primer pairs 5′-AAACGCCAATTCCGACAT-3′ (forward) and 5′-GAAGGACCTCGAGTTACGC-3′ (reverse), and the other set containing primers 5′-CTCTTGGCACCTAGAGGATCT-3′ (forward) and 5′-AAGGTCAGGAGTCACAGCAC-3′ (reverse). Primers for amplification of the human QC transcript were 5′-ACCCTCAATCCCACTGCTAA-3′ (forward) and 5′-CTGGCTTGGAGTCTGAAACA-3′ (reverse). Primers for mouse ACTB were purchased from Qiagen. PCR was performed on a LightCycler instrument (Roche) according to preset protocol and mRNA levels were analyzed by the ΔΔCt method.

### ELISA analysis of total Aβ and pE3-Aβ protein levels

Hemisected brains were homogenized in 2.5 ml of TBS by means of a Precellys homogenizer (Peqlab) followed by sonication for 3 × 10 s. The resulting homogenate was centrifuged at 75,000 × g for 1 h at 4°C. The supernatant was stored at −80°C and Aβ peptides were further extracted with 1 ml of 70% formic acid (FA). The solution was neutralized by addition of a Tris base solution (3.5 M) and further diluted by 6.05 ml of ELISA blocker solution (Pierce). The TBS and FA fractions were subjected to Aβ(×-42) and Aβ(pE3-42) ELISA (IBL International). ELISA analysis was performed according to the manufacturer’s instructions. Following quantification, the Aβ content of both fractions (TBS and FA) was normalized to the brain wet weight.

### Enzyme activity analysis of QCs

QC/isoQC activity was determined using a discontinuous assay, which is based on HPLC-UV. Tissue samples were homogenized in a buffer consisting of 10 mM Tris, 100 mM NaCl, 5 mM EDTA, 0.5% Triton X-100 and 10% Glycerol, pH 7.5, using a Precellys homogenizer (Peqlab). Reaction samples consisted of 50 μM Q-βNA in 25 mM MOPS, pH 7.0, 0.1 mM N-ethylmaleinimide and enzyme solution in a final volume of 1 ml. The reaction temperature was 37°C. Test samples were removed for up to 1 h, and the reaction was stopped by boiling for 5 min. The supernatant was applied to HPLC analyses on a RP18 LiChroCART HPLC Cartridge. The substrate Q-βNA and the pE-βNA were separated by increasing concentration of acetonitrile in water containing 0.1% TFA. The concentration of pE-βNA was determined from a standard curve determined under assay conditions.

### Primary screening of general health and behavior

Semi-quantitative characterization of general health, neurological reflexes and sensory functions was achieved by monthly application of a battery of assays generally referred to as the SHIRPA screening protocol [24]. These consistied in primary screening of muscle and lower motor neuron functions, spinocerebellar, sensory, neuropsychiatric, and autonomic functions. Primary screening provides a behavioral and functional profile by observational assessment, suitable for detecting phenotypes that could interfere with further behavioral assays. Highly standardized primary screens were applied by trained observers blinded to the genotype of the animals and were initiated with observing social behavior in the home cage (home cage observation) subsequently followed by monitoring of undisturbed behavior of single animals in a clear plexiglass arena for 90 s (individual observation). This analysis was followed by a battery of simple behavioral assays characterizing the acoustic startle reflex, hanging behavior, visual placing, falling behavior, righting reflex, postural reflex, negative geotaxis, hanging wire, ear twitch, whiskers twitch, and eye blink, as well as assessment of dysmorphology and body weight [25].

### Automated phenotyping analysis of locomotor activity

Circadian patterns of locomotor activity were assessed using the PhenoMaster system (TSE Systems). Two horizontally staked infrared-sensor frames detected locomotion in the horizontal axis, and rearing events in the vertical axis. Continuous recording of these parameters was carried out simultaneously for all mice in individual observation units (standard type III cages with grid lid) for 136 h or 44 h, starting at 2 p.m. Data was collected automatically with a rate of 100 Hz and stored on a personal computer as a sum over 1 min intervals. The observations took place under a 12-h light/dark-cycle (lights on at 6 a.m.; lights out at 6 p.m.). Animals received water and food *ad libitum* and remained undisturbed by the investigator during observation.

### Acoustic startle response and prepulse inhibition

The experimenter was blinded to the genotype of the animals. Female E85 mice were placed in random order in a startle chamber (TSE Systems) and habituated for 30 s with 68 dB white noise. 10 baseline startle trials with 120 dB white noise were conducted and reactivity was measured in changes of pressure on the floor plate. Baseline determination was followed by prepulse inhibition (PPI) test consisting of 62 randomized trials (10 startle trials: 20 ms of 120 dB white noise; 4 × 10 prepulse + startle trials: 20 ms of white noise preceded 100 ms by 20 ms of 72 dB, 76 dB, 80 dB, or 84 dB white noise; 4 × 3 control trials: 20 ms of 72 dB, 76 dB, 80 dB, or 84 dB white noise followed by 100 ms of 68 dB white noise).

### Immunohistochemical studies

Immersion-fixed brains were dehydrated, embedded in low-melting-point paraffin (DCS Innovative Diagnostic Systems) and sectioned at 8 μm on a rotating microtome. After deparaffinization, sections were incubated with the primary antisera overnight at 4°C. For immunodetection, a biotinylated species-specific IgG secondary antibody was used, followed by signal enhancement with an avidin-biotin-complex (Vectastain ABC Kit; PK-6100; Vector Laboratories) and visualized by peroxidase reactivity (ImmPACT Peroxidase Kit; SK-4105; Vector Laboratories).

In double fluorescent immunolabeling procedures, secondary antibodies were Cy-2- or Cy-3-coupled species-specific IgGs.

In this study Aβ-specific antibody 6E10 (mouse monoclonal; SIG-39320; Covance; 1:10,000 dilution), pE3-Aβ-specific antibodies Abeta-pE3 (rabbit polyclonal; 218003; Synaptic Systems; 1:100,000 dilution) and Abeta-pE3 (mouse monoclonal; 218011; Synaptic Systems; 1:10,000 dilution), QC-specific antibody 1302 (rabbit polyclonal; Probiodrug; 1:1,000 dilution), and human QC-specific antibody 8696 (rabbit polyclonal, Probiodrug; 1:1,000 dilution), glia-specific antibody GFAP (rabbit polyclonal, Z0334; DAKOCytomation; 1:30,000 dilution), activated caspase 3-specific antibody (rabbit polyclonal, 9661, Cell Signaling Technology; dilution 1:100), neuron-specific antibody NeuN (mouse monoclonal; MAB377; Chemicon-Millipore; 1:10,000 dilution), and DARPP32-specific antibody (rabbit monoclonal; 1710–1; Epitomics; 1:10,000 dilution) were used as primary antibodies.

As secondary antibodies, biotinylated mouse-specific IgG (M.O.M. Kit; BMK-2202; Vector Laboratories; 1:500 dilution), biotinylated rabbit-specific IgG (goat, Vector Laboratories; 1:500 dilution), Cy-2-coupled rabbit-specific IgG (goat, Dianova; 1:250 dilution), Cy-3-coupled rabbit-specific IgG (goat, Dianova; 1:250 dilution), Cy-2-coupled mouse-specific IgG (goat, Dianova; 1:250 dilution), and Cy-3-coupled mouse-specific IgG (goat, Dianova; 1:250 dilution) were used.

### Semi-automated quantification with CellProfiler

For quantification, 8 μm coronal sections, of the right hemisphere, corresponding to stereotaxic levels bregma 0.14 mm to 0.38 mm (as defined in Paxinos and Franklin [26]) were used for analysis. To quantify pE3-Aβ positive cells, images including all stained striatal cells of pE3-Aβ stained slides were taken. To quantify neuronal numbers, parallel sections were stained with NeuN and a region of interest (ROI) was defined. Images were taken with the Picture Frame Application 2.3 (Optronics) using a Nikon Eclipse 80i microscope (Nikon Instruments) with a MicroFIRE2.3A camera (Optronics).

To count the number of stained cells, CellProfiler (CP) (r10997) software was used, which enables simultaneous high throughput measuring of size, shape, intensity and texture of cells [27]. A detailed protocol, definition of the ROI, both used ‘pipelines’, and raw data examples are provided as Appendix 1. Briefly, every image was inverted, converted from RGB to grayscale and stained cells were identified by contrast, shape, and size.

## Results

### Transgenic integration

**Figure 1:**
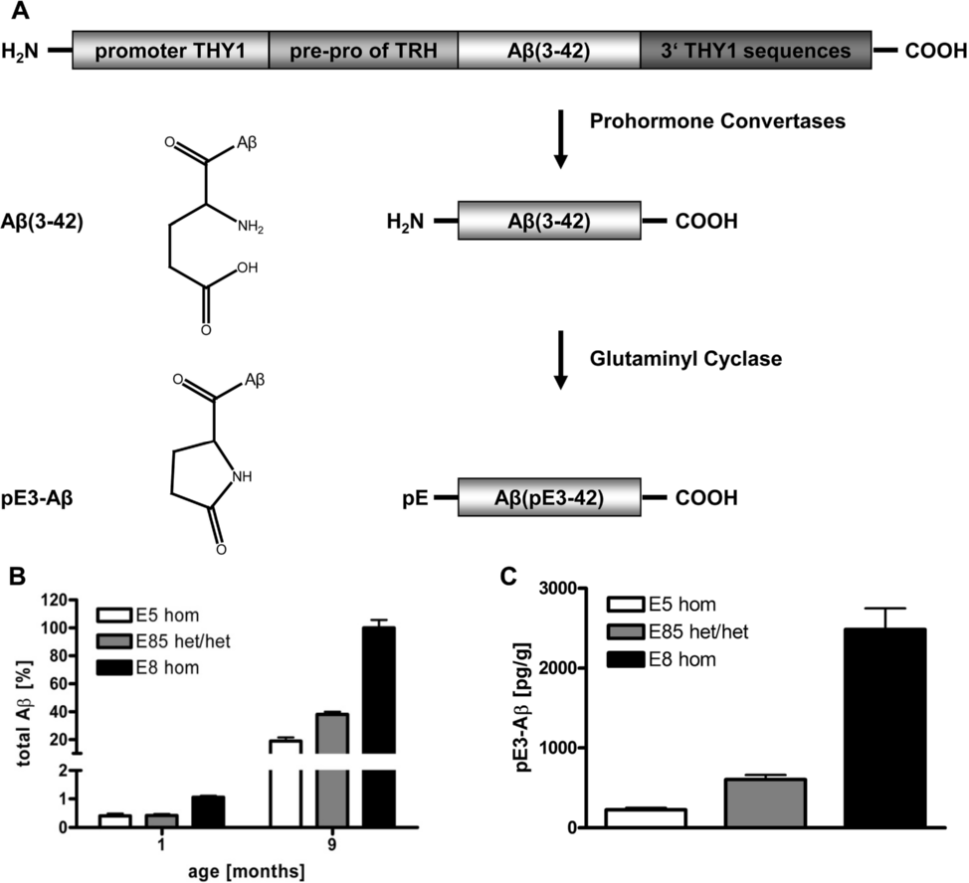
**Construct expression and processing.** In the construct of ETNA Aβ(3–42) is fused to pre-pro-TRH for product liberation within the secretory pathway. After prohormone convertase cleavage the N-terminally truncated Aβ peptide is transported into the trans-Golgi and secretory vesicles, where the N-terminus is available to QC for cyclization **(A)**; adapted from Alexandru et al., 2011. Quantification of protein levels by ELISA of ETNA brains revealed highest total Aβ levels for hom E8, lowest for hom E5 and intermediate for het/het E85 at two different ages **(B)**; total Aβ values expressed as percentage of hom E8 at the age of 9 months; data represent means ± SEM; n ≥ 5 animals per genotype. These protein level relations between the three lines are reflected by similar relations of pE3-Aβ levels at the age of 9 months **(C)**; data represent means ± SEM; n ≥ 5 animals per genotype.

Several tg mouse lines were generated and examined alone or in intercrosses in order to characterize QC-dependent pE3-Aβ formation and neurotoxicity *in vivo*. The expression cassettes of ETNA lines consist of the pre-pro-sequence of murine TRH fused to the coding sequence of human Aβ(3–42) (Figure 1A). Processing of the TRH sequence in the secretory pathway of neurons is facilitated by signal peptidases and PCs [28] leading to the preferential liberation of Aβ(3–42) within the trans-Golgi network and secretory granules. Two independent tg lines (E5 and E8) were established to examine transgene dosage effects. By intercrossing both lines, double het/het animals were generated. These animals were termed E85 and examined in order to minimize data misinterpretation due to positional effects based on chromosomal transgene insertion.

In transgene integration mapping experiments for line E8, the 5′ flanking region of the expression cassette was identified as part of a DNA repeat element, presumably on chromosome 6. This element is repeated eight times and therefore the exact localization of the expression cassette remains unknown. Attempts to isolate the 3′-flanking regions were not successful.

In line E5, the transgene cassette has integrated as multimer in a head-to-head orientation. The mapping experiments did not deliver any flanking chromosomal regions. However, the head-to-head orientation of the cassette allows differentiation of the E5 and E8 tg alleles in PCR-based genotyping assays.

Mapping experiments for mouse line hQC demonstrated that a 3′- integration of the expression cassette occurred on chromosome 13 (map position 89.0 Mbp; NCBI Build 36) in intron 1 of the mouse Edil3 gene (EGF-like repeats and discoidin I-like domains 3). PCR reactions on homozygote DNA with primer sets binding to regions upstream of the integration site showed that the integration event deleted a DNA region of about 50 kb. Hence, integration of the expression cassette removed the first coding exon of Edil3 and hom animals are devoid of Edil3 gene function.

### Construct expression and processing

Transgene expression was quantified using qRT-PCR analysis in those lines originating from the carefully selected founder lines. E5 and E8 show similar mRNA levels, with E5 expressing about 90% of the transgene compared to E8 (data not shown).

ELISA analysis of protein levels revealed substantial amounts of total Aβ in E5, E85, and E8. At both 1 and 9 months of age, highest levels were detected in E8 and lowest in E5, while levels in E85 were intermediate (Figure 1B). Total Aβ levels detected by ELISA include all forms endogenic Aβ, the truncated Aβ(3–42) construct, unprocessed mTRH prepro-Aβ(3–42), and also any partially processed precursor molecules. Concentrations up to 2.5 mg/g were detected in the brains of hom E8, although the exact amount of present Aβ could not be determined.

Showing distinct pyroglutamyl modification of the N-terminally truncated Aβ(3–42) peptide, significant levels of pE3-Aβ were detected in all 3 lines. At the age of 9 months these pE3-Aβ levels reflected a similar distribution pattern among genotypes as detected for total Aβ levels (Figure 1C).

### General health and behavioral phenotype

Monitoring of general health revealed an average litter size of all ETNA mouse lines (E5, E8, and E85) in accordance with the expectation from a C56BL/6 inbred background. Analysis of genotypes revealed a nearly Mendelian ratio of homozygous (hom), het, and wt genotypes (data not shown).

**Figure 2:**
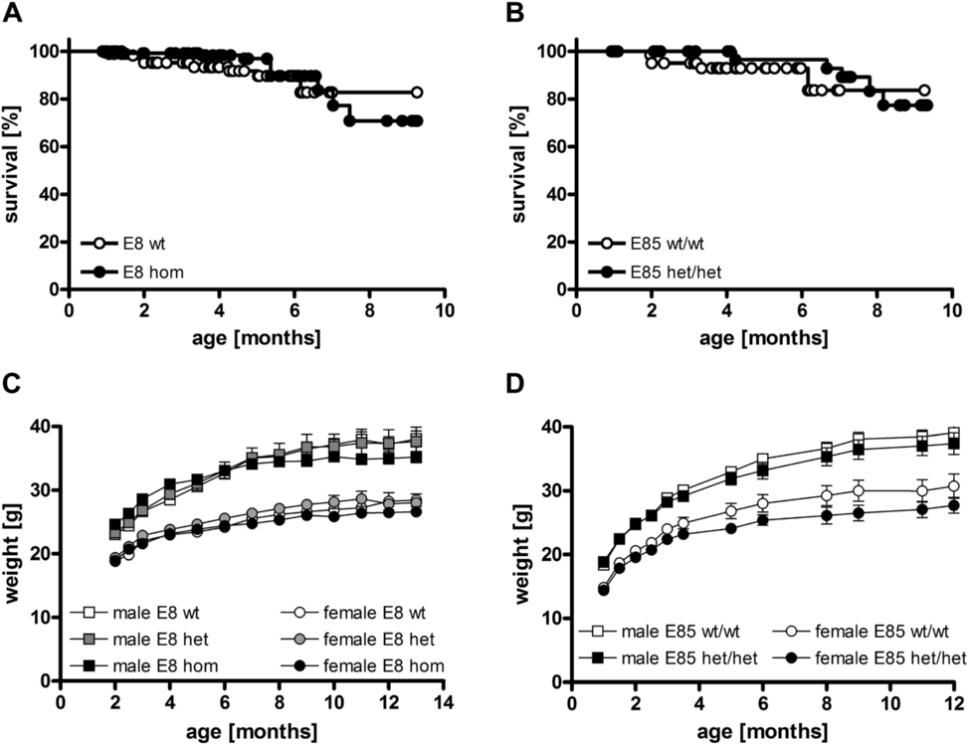
**General health.** Monitoring of survival rates up to the age of 10 months revealed no significant differences between hom E8 **(A)** or het/het E85 mice **(B)** compared to wt animals, respectively; dots represent dead or censored animals; data were analyzed by logrank test of survival curves; p > 0.05; n ≥ 50 animals per group. Monitoring of bodyweight revealed a moderate, but not significantly reduced gain of weight for hom E8 **(C)**; data of each gender were analyzed by two-way ANOVA for repeated measurements followed by Bonferroni’s *post hoc* test (factor genotype, n.s. p > 0.05) and represent means ± SEM; n = 4–10 animals per group. A similar reduction of weight gain was observed in het/het E85 **(D)** compared to wt animals; data of each gender were analyzed separately by two-way ANOVA for repeated measurements followed by Bonferroni’s *post hoc* test (factor genotype, n.s. p > 0.05) and represent means ± SEM; n = 10–16 animals per group.

Analysis of survival revealed no significant differences between genotype groups for hom E8 (Figure 2A) and het/het E85 (Figure 2B) mice until the age of 10 months, but showed a trend for higher mortality in both E8 and E85.

Measurement of weight revealed no significant differences between hom E8 (Figure 2C) or het/het E85 (Figure 2D) mice and wt littermates, respectively.

Monthly monitoring of general health, neurological reflexes and sensory functions in the primary screening showed no obvious alterations, with the exception of the acoustic startle reflex (see section acoustic reactivity and sensorimotor gating).

Various tests for basal physiological responses as well as in-depth characterization of emotionality, cognition, and pain perception revealed no significant differences (data not shown).

### Locomotor activity

To analyze the circadian pattern of various behavioral parameters, ETNA mice were monitored using the automated PhenoMaster system. In this system, food and water consumption, as well as locomotor and rearing activity were recorded. None of the tested animals showed evidence of altered feeding or drinking behavior (data not shown).

**Figure 3:**
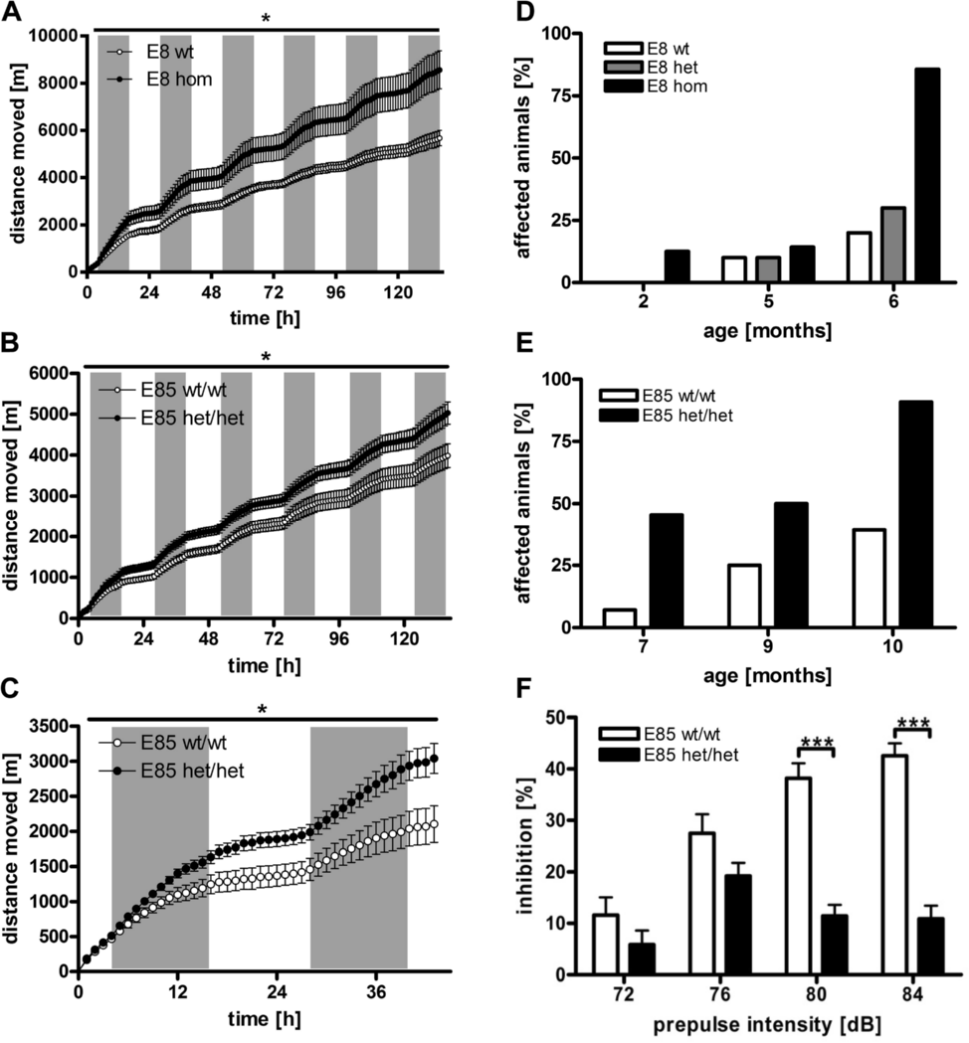
**Behavioral phenotype.** Automated recording of locomotion increased activity of hom E8 animals at the age of 10 months **(A)**; gray bars indicate dark phases; dots show circadian pattern of cumulative distance moved; data were analyzed by two-way ANOVA for repeated measurements followed by Bonferroni’s *post hoc* test (factor genotype, * p = 0.032) and represent means ± SEM; n ≥ 4 animals per genotype. This hyperactivity was confirmed in het/het E85 mice at the age of 6 months **(B)**; data were analyzed by two-way ANOVA for repeated measurements followed by Bonferroni’s *post hoc* test (factor genotype, * p = 0.012) and represent means ± SEM; n = 12 animals per genotype. At the age of 8 months in het/het E85 mice, the significantly increased activity is already detectable after 44 h of measurement **(C)**; data were analyzed by two-way ANOVA for repeated measurements followed by Bonferroni’s *post hoc* test (factor genotype, * p = 0.017) and represent means ± SEM; n ≥ 10 animals per genotype. Monthly primary screening revealed a progressive decrease of reactivity on a given acoustic signal, affecting ~90% hom E8 animals at the age of 6 months **(D)**; bars show percentage of affected animals; n ≥ 7 animals per genotype; only males were used. In het/het E85 animals this behavioral phenotype was delayed, affecting ~90% at the age of 10 months **(E)**; bars show percentage of affected animals; n ≥ 22 animals per genotype. Analysis of het/het E85 animals at the age of 7 months, revealed a decrease of PPI of the auditory startle reflex across 72–84 dB prepulse intensity **(F)**; data were analyzed by two-way repeated-measures ANOVA followed by Bonferroni’s *post-hoc* test and represent means ± SEM; *** p < 0.001 n = 9–10 animals per genotype.

In hom E8 mice aged 10 months, measurement of total distance moved within 136 h showed significantly increased locomotor activity compared to wt littermates (Figure 3A).

For a more detailed analysis, the locomotor activity of het/het E85 mice was regularly monitored. Beginning with an age of 4 months het/het E85 mice showed progressive hyperactivity alterations (data not shown). This elevated activity is significantly increased starting at the age of 6 months in het/het E85 mice (Figure 3B). At the age of 8 months the significant increase in total distance moved of het/het E85 animals is detectable after only 44 h of recording (Figure 3C).

### Acoustic reactivity and sensorimotor gating

Monitoring of E8 mice in the primary screening revealed an impaired reaction to a given acoustic signal beginning at the age of 2 months affecting 90% of hom E8 animals at the age of 6 months (Figure 3D).

For a more detailed analysis het/het E85 mice were monitored monthly at the age of 2 to 10 months in the primary screening, which showed significantly increased percentages of affected animals compared to wt littermates (paired t-test of 9 time points, ** p = 0.007, n ≥ 22 animals per genotype).

Importantly, 50% of wt C57BL/6 mice showed reduced reactivity at the age of 10 months. This is attributable to an age-related hearing loss mutation in the *Cdh23*^*ahl*^ gene [29,30]. Nonetheless, more than 90% of het/het E85 animals were affected, arguing for impaired sensorimotor gating in these animals (Figure 3E).

To address the cause of reduced acoustic reactivity, PPI of het/het E85 mice was analyzed. The significant loss of PPI compared to wt animals indicates impaired sensorimotor gating (Figure 3F).

### Expression of the transgenic product Aβ(3–42)

To identify the expression pattern of the tg product Aβ(3–42), coronal E5 and E8 brain sections from different brain regions and at different ages were stained with the anti-Aβ antibody 6E10. This histopathological analysis revealed an early expression of the construct in different brain regions, with most prominent expression in the striatum.

High immunoreactivity was also detected in amygdala (Additional file 1: Figure S1B) and single 6E10 positive cells were found in cortex and brainstem (Additional file 1: Figure S1D, E) up to the age of 9 months in ETNA animals.

**Figure 4:**
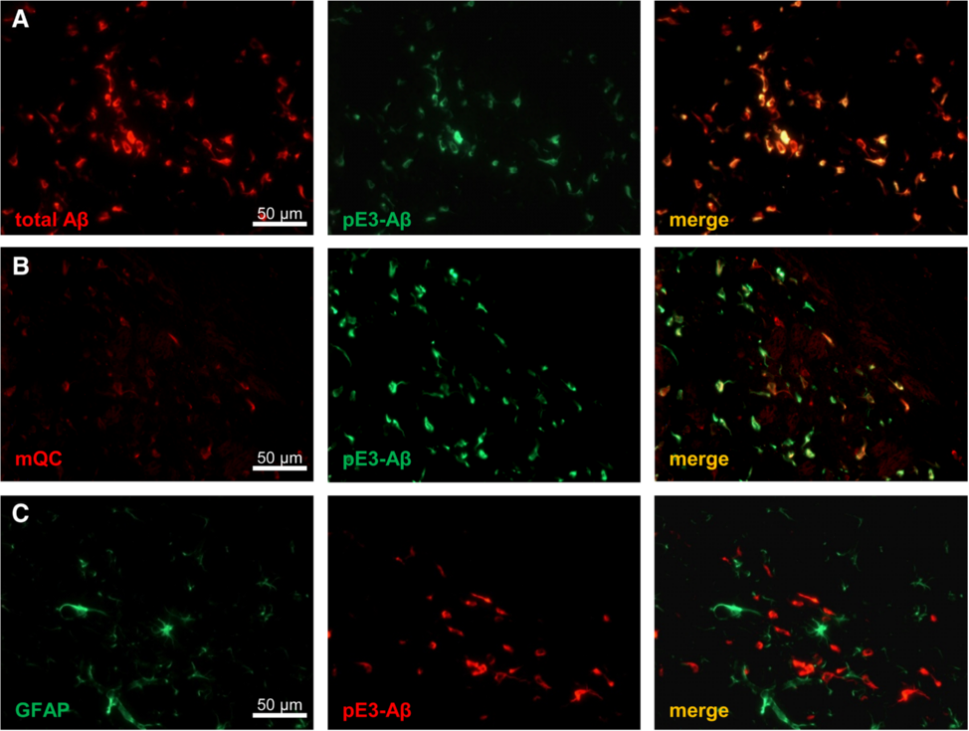
**Striatal (co-)localizations of Aβ, pE3-Aβ, QC and astroglia in lateral striatum.** Double immunofluorescent labeling of total Aβ (red) and pE3-Aβ (green) revealed intraneuronal colocalization of tg product and pE-modified Aβ in the lateral striatum of hom E8 animals **(A)**. Double immunofluorescent labeling of the lateral striatum showed mQC-specific immunoreactivity (red), which is colocalized with pE3-Aβ (green) in hom E8 animals **(B)**. Double immunofluorescent labeling revealed increased GFAP reactivity (green) of activated astroglia in the vicinity of pE3-Aβ positive cells (red) in the lateral striatum of hom E8 animals **(C)**.

In all ETNA lines, the striatal 6E10 signal is intraneuronally colocalized with pE3-Aβ reactivity (Figure 4A). 6E10 reactivity was observed in the pyramidal cell layer of the hippocampus of ETNA mice Additional file 1: Figure S1C) but up to the age of 9 months (latest time point of analysis) no pE3-Aβ formation was found (data not shown).

Double immunofluorescent labeling of striatal sections revealed mouse QC (mQC)-specific immunoreactivity in pE3-Aβ positive cells, suggesting QC-dependent pE3-Aβ formation (Figure 4B).

To further analyze neuropathology, GFAP staining was performed and revealed activated astroglia in the vicinity of pE3-Aβ positive neurons, indicating neuroinflammatory processes in the lateral striatum of ETNA animals (Figure 4C).

### Kinetics

To analyze the kinetics of neuropathology, protein levels were quantified by ELISA and the striatum of E5, E85, and E8 mice were stained with markers for Aβ species, neuroinflammation, neurodegeneration, and dopamine- and cAMP-regulated phosphoprotein of 32 kDa (DARPP-32) across age. In hom E8 animals, progressive pE3-Aβ formation and neurodegenerative processes are observed from the age of 3 months, while E5 and E85 mice show delayed kinetics (data not shown).

**Figure 5:**
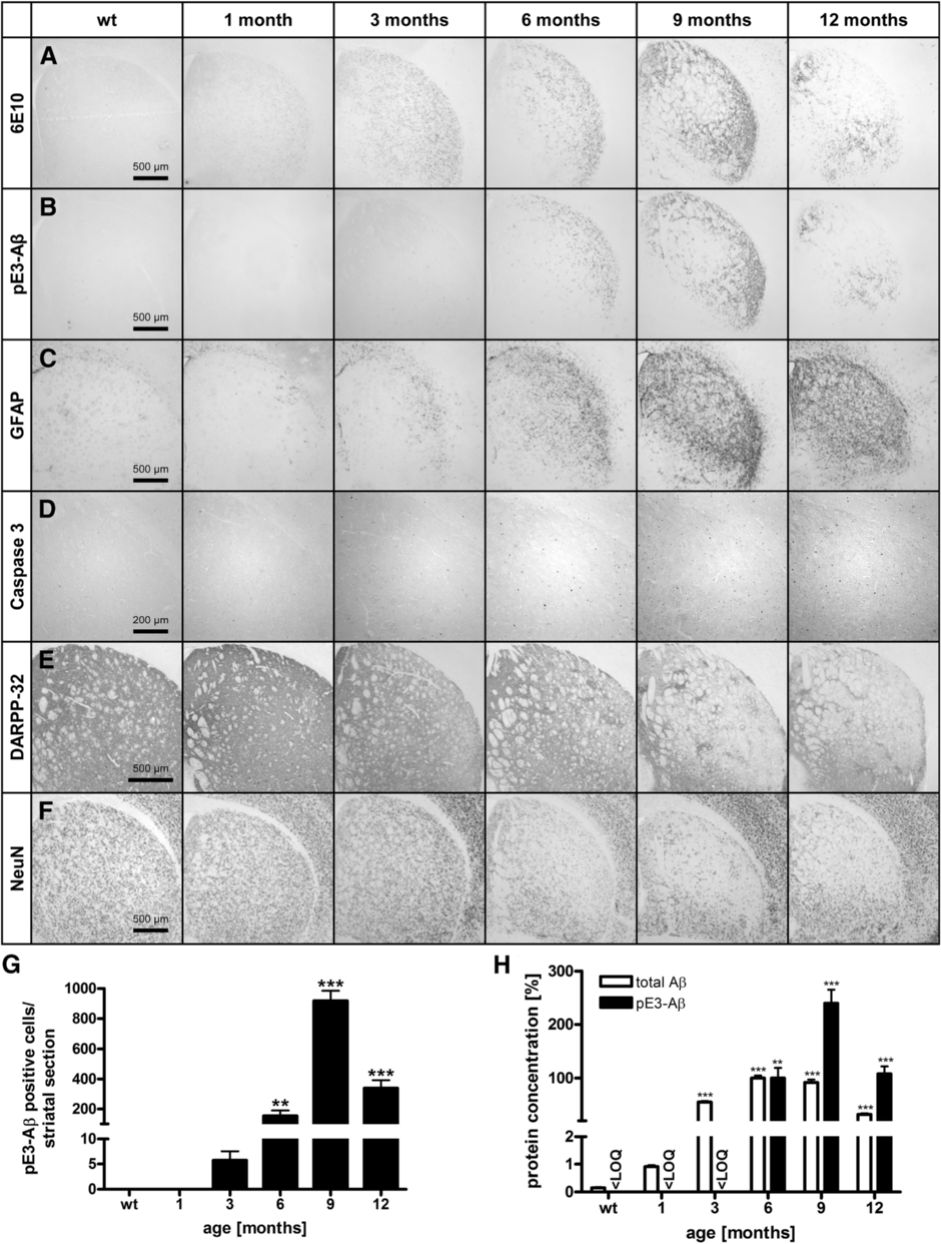
**Kinetics of homozygous E8 mice.** Immunohistochemical analysis of hom E8 animals from the age of 1–12 months compared to wt animals **(A-F)**. Staining with 6E10 revealed progressive increase of total Aβ immunoreactivity from the age of 1–9 months with a decline at 12 months **(A)**. Increase of pE3-Aβ reactivity from 3–9 months and decline at 12 months **(B)** is accompanied by GFAP reactivity **(C)**. Apoptotic processes are indicated provided by progressive caspase 3 activity beginning at the age of 3 months **(D)**. The lateral striatum shows a progressive decrease of DARPP-32 reactivity of neuropil from the age of 6 months **(E)**. The different processes result in decreased NeuN positive cells of E8 animals indicating cell loss beginning in at the age of 6 months **(F)**. Quantification of pE3-Aβ positive cells in the striatum of hom E8 animals revealed exponential increase from 3–9 months and declining numbers at the age of 12 months to the level of 6 months **(G)**; data were analyzed by one-way ANOVA followed by Newman-Keuls *post-hoc* test and represent means ± SEM; ** p < 0.01; *** p < 0.001; n = 4–8 animals per group. Quantification of protein levels by ELISA of hom E8 brains revealed an progressing increase of total Aβ until the age of 6 months; pE3-Aβ protein levels are detectable from the age of 6, reaching its maximum at the age of 9 and declining at 12 months **(H)**; protein values are expressed as percentage of hom E8 brains at the age of 6 months; data were analyzed by one-way ANOVA followed by Newman-Keuls *post-hoc* test and represent means ± SEM; ** p < 0.01; *** p < 0.001; n = 3–9 animals per group;<LOQ (below level of quantification).

Brains of hom E8 animals were analyzed in more detail at the age of 1, 3, 6, 9, and 12 months and compared to wt animals (Figure 5).

Expression of the Aβ construct in the striatum can already be visualized starting at the age of 1 month with increasing signal up to 9 months (Figure 5A), whilst pE3-Aβ is detected from 3 months of age (Figure 5B). Quantification of pE3-Aβ positive cells revealed an exponential increase from 3 to 9 months (Figure 5G).

Staining with GFAP revealed activated astroglia in regional and temporal correlation of pE3-Aβ positive cells until the age of 9 months, while at 12 months the whole striatum shows extensive neuroinflammation (Figure 5C).

Activated caspase 3 signal progressing from the age of 3 months suggests pE3-Aβ-associated apoptotic processes in the lateral striatum of hom E8 animals (Figure 5D).

Progressive decrease of DARPP-32 reactivity of neuropil in the same region was detected starting at the age of 6 months (Figure 5E), while intraneuronal immunoreactivity seemed to be unaffected (data not shown).

These neurodegenerative processes ultimately resulted in decreased numbers of NeuN positive cells indicating striatal cell loss beginning in at the age of 6 months (Figure 5F).

Quantification of protein levels by ELISA of whole E8 brain hemispheres revealed weak construct expression in juvenile animals, while from 3 to 12 months total Aβ is comparatively robust (Figure 5H; white bars).

Levels of pE3-Aβ are detectable from the age of 6 months rising until 9 months (Figure 5H; black bars).

The protein levels are in line with histopathological analysis of striatum, highlighting the striatum as the region with the strongest alterations in the ETNA mouse model.

Reductions of protein levels and immunoreactive cells of total Aβ and pE3-Aβ at 12 months seem to be caused by the lack of construct-expressing and pE3-Aβ-forming neurons in the degenerated striatum of homozygous E8 animals.

### QC overexpression

To analyze the influence of QC on pE3-Aβ and neuropathology, E8 and E85 animals were intercrossed with mice overexpressing human QC (hQC) generating ETNA-hQC mice.

**Figure 6:**
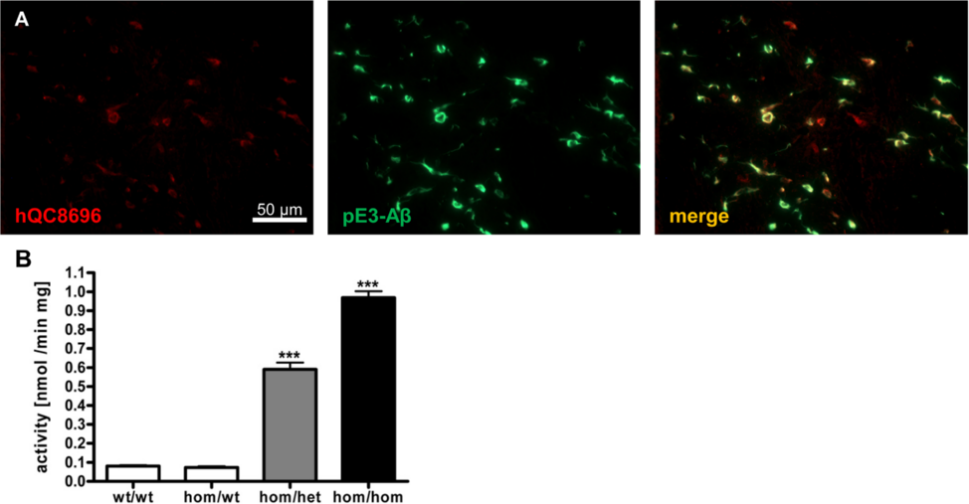
**QC overexpression.** Double immunofluorescent labeling of hom/hom E8-hQC animals revealed human QC specific immunoreactivity (red) colocalized with pE3-Aβ (green) in the striatum **(A)**. Analysis of QC activity at the age of 6 months revealed an indistinguishable activity for ETNA animals compared to wt animals and a ~60-fold increase for het and ~100-fold increase for hom overexpression of hQC **(B)**; data were analyzed by one-way ANOVA followed by Newman-Keuls *post-hoc* test and represent means ± SEM; *** p < 0.01; n = 2–12 animals per group.

Staining of striatal brain sections of E8-hQC animals with specific hQC antibody confirmed intraneuronal co-expression of hQC and pE3-Aβ (Figure 6A).

While endogenous QC-activity is constant in aging ETNA mice and shows the same levels as wt control animals (data not shown), het overexpression of hQC leads to a ~5 fold increase and hom overexpression to an ~10 fold increase of QC activity in the brains of ETNA animals (Figure 6B).

ETNA hom and ETNA-hQC hom/wt showed indistinguishable total Aβ levels, pE3-Aβ levels, and striatal neuropathology confirming that the genetic background of hQC wt animals had no influence on pE3-Aβ formation or neurodegeneration (data not shown).

### Influence of hQC overexpression on pE-Aβ formation

**Figure 7:**
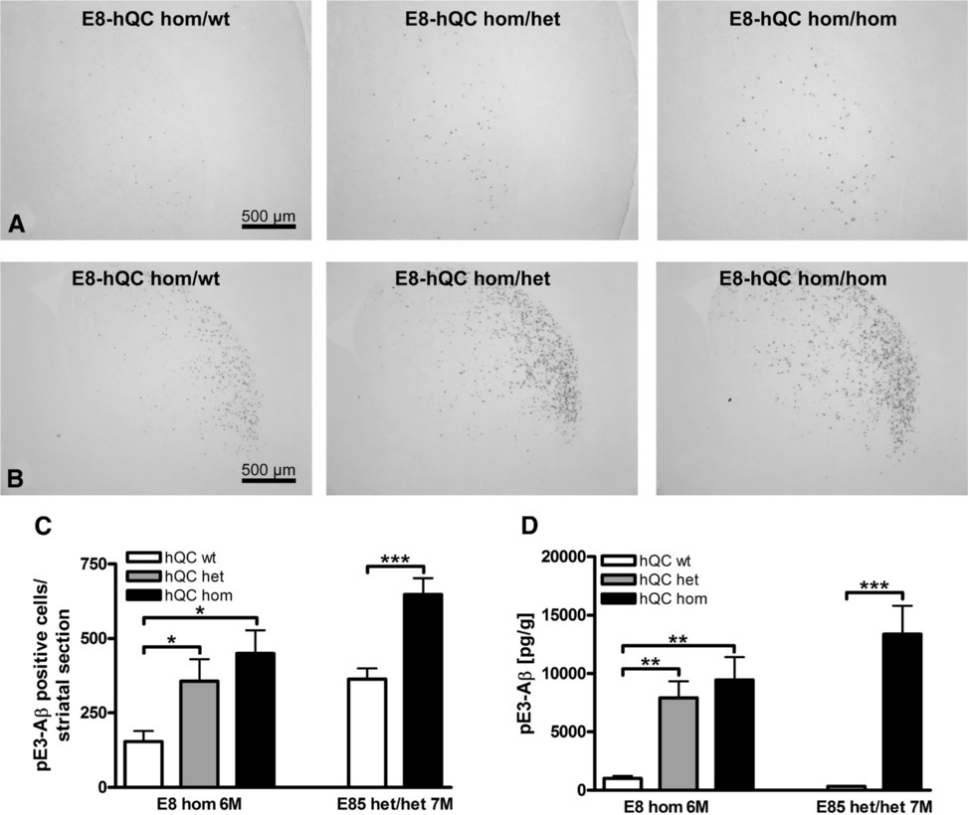
**Influence of QC overexpression on pE3-Aβ formation.** Staining of striatal sections of transgenic E8-hQC mice demonstrated an increase of pE3-Aβ positive cells by het and hom hQC overexpression, respectively at the age of 3 months **(A)** and 6 months **(B)**. Quantification of these pE3-Aβ positive cells revealed a significant increase for het and hom overexpression of hQC in homs E8 animals at the age of 6 months and in het/het E85 animals at the age of 7 months **(C)**; E8 were analyzed by one-way ANOVA followed by Newman-Keuls *post-hoc* test; E85 were analyzed by unpaired t-test; data represent means ± SEM; * p < 0.05; *** p < 0.001; n = 7–11 animals per group. Quantification of pE3-Aβ levels by ELISA revealed a significant increase for het and hom overexpression of hQC in hom E8 brains at the age of 6 months and in het/het E85 brains at the age of 7 months **(D)**; E8 were analyzed by one-way ANOVA followed by Newman-Keuls *post-hoc* test; E85 were analyzed by unpaired t-test; data represent means ± SEM; ** p < 0.01; *** p < 0.001; n = 5–11 animals per group.

Already at the age of 3 months, enhancing QC activity in hom E8 animals lead to a gene dosage-dependent increase of striatal pE3-Aβ positive cells (Figure 7A) which was obvious at 6 months of age (Figure 7B).

As expected, quantification of pE3-Aβ positive cells revealed a significant increase in E8-hQC and E85-hQC mice (Figure 7C).

Quantification of pE3-Aβ levels by ELISA revealed similar distribution, but E85-hQC animals showed a more than 10fold increase of pE3-Aβ protein value compared to E85 mice (Figure 7D).

Overexpression of hQC had no influence on total Aβ levels detected by ELISA in both ETNA-hQC lines (data not shown) arguing for QC-dependent pE3-Aβ formation in the ETNA mouse model.

### Influence of hQC overexpression on neuropathology

**Figure 8:**
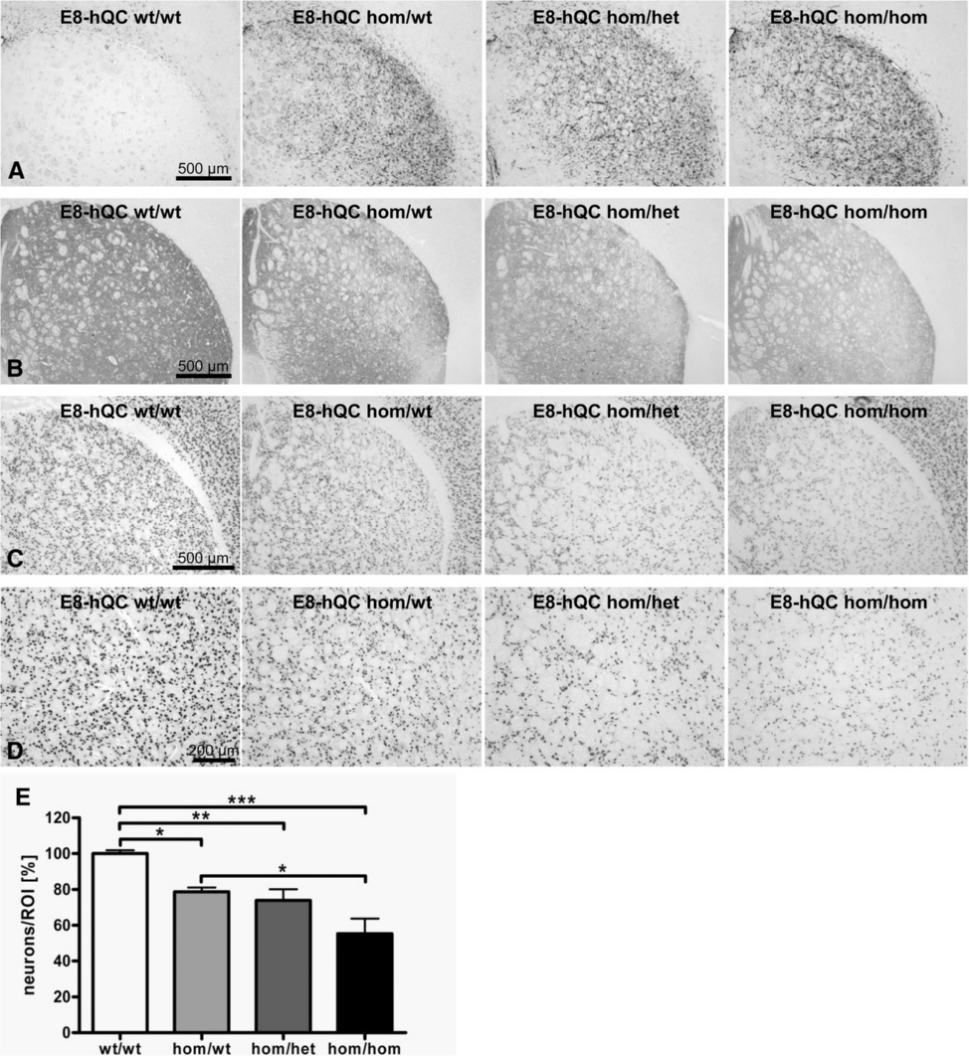
**Influence of QC overexpression on neuropathology.** Immunohistochemical staining of 6 month old double tg E8-hQC mice revealed hQC dependent increase of GFAP reactivity of activated astroglia in the striatum **(A)**. Staining of 6 month old E8-hQC mice revealed hQC dependent decrease of DARPP-32 reactivity of striatal neuropil **(B)**. These neuroinflammatory and degenerative processes resulted in hQC dependent decrease of NeuN positive cells demonstrating striatal cell loss beginning in at the age of 6 months **(C)**. Quantification of neuronal numbers in a defined region of interest (ROI) in the basolateral striatum **(D)** of E8-hQC mice revealed a neuronal loss of 21% for hom/wt, 26% for hom/het, and 45% for hom/hom **(E)**; neuronal numbers expressed as percentage of wt; data were analyzed by one-way ANOVA followed by Newman-Keuls *post-hoc* test and represent means ± SEM; * p < 0.05; ** p < 0.01; *** p < 0.001 n = 5–8 animals per genotype.

To analyze the influence of hQC overexpression on neuropathology, double tg E8-hQC mice at the age of 6 months were stained with neurodegenerative and neuroinflammatory markers and striatal cell loss was quantified (Figure 8).

Although hom E8 animals showed obvious striatal neuroinflammation compared to wt animals, GFAP reactivity seemed to be enhanced by overexpression of hQC (Figure 8A).

Low DARPP-32 reactivity in striatum of homozygous E8 brain sections was further decreased by overexpression of hQC (Figure 8B).

Neuron-specific staining with NeuN-antibody of E8-hQC mice showed hQC dependent decrease of immunoreactivity (Figure 8C). To further analyze the influence of hQC overexpression on neurodegeneration, a region of interest (ROI) was defined in the lateral striatum (Figure 8D) and neuronal numbers were quantified.

Quantification of neurons in the ROI of 6 months old E8-hQC mice revealed a significant increase of neuronal loss from 21% for hom/wt to 45% for hom/hom compared to wt animals (Figure 8E).

## Discussion

Numerous studies support a gain of toxic function of Aβ due to post-translational modifications such as isoaspartate formation, nitrosylation [31], and formation of pyroglutamate [10,11,30,32]. In order to assess the pathological consequence of enhanced pE-Aβ *in vivo*, several tg mouse models expressing truncated human Aβ with an N-terminal Q to E substitution have been generated [17-19]. Although these models display differences in terms of behavior and neuronal loss, they commonly suggested that an increase of pE-Aβ enhances neuronal dysfunction. With regard to TBA2 and TBA2.1 mouse models, the ETNA gene product is the same. However, pE3-Aβ formation in ETNA is not accelerated by an exchange of E by Q at position 3 of Aβ. As shown in previous studies, the N-terminal E residues represent worse substrates for QC (and isoQC) compared to Q, and E residues should not cyclize spontaneously considering a half-life of 10–40 years *in vivo*[2,3]. In accordance with such slower formation of pE-Aβ(3–42) also *in vivo*, we observed the behavioral changes and neuronal loss at a later time point compared to TBA2.1.

Furthermore, in ETNA the tg product, Aβ(3–42), is mainly expressed in the lateral striatum compared to the hippocampal and brainstem expression in TBA2.1. In this brain region ETNA animals show progressive intraneuronal pE3-Aβ formation associated with neuroinflammatory, apoptotic, and neurodegenerative processes, including but not limited to a reduced DARPP-32 immunoreactivity. The behavioral phenotype of single tg ETNA mice, hyperactivity and impaired acoustic sensorimotor gating, is associated with striatum the brain region with distinct neuropathology.

Key feature of the neuropathological process is a progressive site-specific expression of pE3-Aβ, which is associated with increased caspase 3 and GFAP expression, ultimately resulting in significant cell loss of medium-sized spiny neurons (MSN), the major cell type in the striatum [33].

Interestingly, TBA2.1 mice have also shown expression of the Aβ(Q3-42) construct in the striatum and hippocampus. Only in the latter brain region, pE3-Aβ formation and neurodegeneration is observed in these animals [17]. ETNA mice, which use the same Thy1.2 promoter and expression cassette and only differ in one amino acid, express the Aβ(E3-42) construct in the same regions, but surprisingly pE3-Aβ formation and neurodegeneration mainly occurs in the striatum. In both animal models, TBA2.1 and ETNA, neuronal loss is exclusively observed in vicinity of pE3-Aβ, arguing for pE3-Aβ as the neurotoxic Aβ species.

Compared to other unmodified Aβ, pE3-Aβ had been shown to accelerate accumulation of several Aβ species, serving as seed of Aβ aggregation [15-17]. Although, the presence of other Aβ species might contribute for the phenotype of ETNA animals, the properties of pE3-Aβ and the spatio-temporal correlation with neuropathology argue for pE3-Aβ as crucial factor. In summary, the presence of pE3-Aβ seems to trigger the coaggregation and to influence the neurotoxic properties of other Aβ species.

Despite for the generation of TBA mice the same Thy1.2 promoter was used as for ETNA mice, in all models a different brain region showed the most expression of the tg construct. In TBA2 the most tg expression was found in the cerebellum [18], TBA2.1 mainly expressed Aβ in the hippocampus [17], and ETNA mice showed most expression in the striatum. The different insertion sites of the tg construct into the genome might have led to the regional variation of expression intensity, but the exact mechanism remains unclear.

Also, the kinetics of pE3-42 deposition and neuropathology implies a crucial role of the modified peptide for neuronal loss. Already at the age of 1 month (first time point of analysis), hom E8 show expression of the truncated Aβ(3–42) construct in the lateral striatum and severe Aβ protein levels of brain homogenates were detected by ELISA. With 3 months of age Aβ expression of hom E8 animals is increasing, reaching a plateau at 6 months. At this age, immunohistochemical stainings then revealed exponential pE3-Aβ formation being associated with immunoreactivity specific for activated astroglia, increased caspase 3 activity, decrease of DARPP-32 expression, and blunted NeuN immunoreactivity, which altogether describe neuroinflammatory and apoptotic neurodegenerative processes.

In temporal correlation of prominent pE3-Aβ formation first behavioral alterations were detected in single tg ETNA animals and at the age of 6 months 90% of hom E8 animals showed decreased reactivity on a given acoustic signal. These animals showed obvious neuronal loss in the lateral striatum at the age of 9 months and hyperactivity was detected at the age of 10 months.

In het/het E85 animals the same chronology of neuropathology and behavioral phenotype was observed with a delay of 2–4 months.

Moreover, overexpression of hQC has been described to enhance pE3-Aβ dependent pathology and behavior in 5XFAD-hQC mice [34]. To further analyze the role of QC on pE3-Aβ formation and neuropathology, E8 and E85 animals were intercrossed with human QC overexpressing mice to generate double tg ETNA-hQC mice.

These mice showed elevated pE3-Aβ levels and increased neuropathology compared to single tg ETNA mice providing evidence for QC-dependent formation of neurotoxic pE3-Aβ *in vivo*. Overexpression of hQC had no influence on total Aβ levels detected by ELISA in both ETNA-hQC lines (E8-hQC and E85-hQC), but increased pE3-Aβ levels and numbers of pE3-Aβ positive cells in the lateral striatum. Double tg E8-hQC animals showed hQC gene dose-dependent increase of GFAP and decrease of DARPP-32 reactivity in the striatum at 6 months of age. Neuronal numbers were quantified in the basolateral striatum of these animals and revealed a significantly increased neuronal loss of 45% for hom/hom E8-hQC animals compared to 21% for hom/wt E8-hQC animals with hQC wt expression. Enhanced pE3-Aβ formation and pathology in hQC overexpressing animals provide direct evidence for a QC-driven process, limited by the available amount of QC.

The striatal pathology of ETNA contrasts with other mouse models such as TBA2.1 or hAPP tg mice [35-37]. In most of these models, hippocampal (and cortical) degeneration has been observed. ETNA mice display only weak expression of the construct in the pyramidal cell layer of the hippocampus (Additional file 1: Figure S1C), from the age of 3 months and no pE3-Aβ formation is observed up to the age of 9 months in E5, E8, and E85 animals (data not shown).

In accordance with the striatal neuronal loss, sensorimotor gating impairments, similar to striatal alterations in HD, have been observed in ETNA mice [38]. Likewise, in mice with a genetic deletion of DARPP-32 or with point mutations in phosphorylation sites of DARPP-32, the effects of stimulating drugs on sensorimotor gating were strongly attenuated [39].

Impairment of acoustic sensorimotor gating is associated with striatal neurodegeneration occurring at the sites of pE3-Aβ expression and diminished DARPP-32 immunoreactivity.

Similarly, the home cage hyperactivity displayed by single tg ETNA mice is likely to be a consequence of the underlying striatal pathology, possibly caused by a loss of GABAergic inhibitory neurotransmission resulting in a disinhibition of motor function.

While hyperkinetic movement disorders represent an important subgroup of neurodegenerative disorders, not all of those affect the striatum [40]. Among them, HD shows the highest incidence and has been associated with disturbed dopaminergic as well as downstream DARPP-32 neurotransmission [41,42]. A loss of inhibitory GABAergic MSNs represents a key feature of the disease and is causal for hyperactivity [43]. In addition to their relevance for pE3-Aβ toxicity in AD, ETNA lines might model selected aspects of HD.

## Conclusions

Taken together, ETNA is a new mouse model for Aβ-toxicity and neuronal loss, associated by a region specific behavioral phenotype.

The immunohistological characterization, analysis of protein levels, and behavioral findings of all ETNA lines strongly support a crucial role of QC-catalyzed pE3-Aβ for induction and progression of striatal neuronal loss. Therefore, the model might be also of interest for investigation of the role of striatal factors such as DARPP-32 or others in neuronal degeneration in HD as well as for testing of neuroprotective strategies targeting striatal degeneration.

## Appendix 1

### Construct Expression in ETNA animals

To identify the expression pattern of the tg product Aβ(3–42), coronal brain sections of E5, E8 and E85 from different brain regions and at different ages were stained with the anti-Aβ antibody 6E10. In all lines, highest immunoreactivity was found in the striatum (Additional file 1: Figure S1A). Obvious immunoreactivity was also detected in amygdala (Additional file 1: Figure S1B) and single 6E10 positive cells were found in cortex and brainstem (Additional file 1: Figure S1D, E) up to the age of 9 months. 6E10 reactivity in the pyramidal cell layer of the hippocampus of ETNA was observed (Additional file 1: Figure S1C), but up to the age of 9 months (latest time point of analysis) no pE3-Aβ formation was found (data not shown).

### Protocol of pE3-Aβ cell and neuronal quantification

For quantification 8 μm coronal sections, of the right hemisphere, corresponding to stereotaxic levels bregma 0.14 mm to 0.38 mm (as defined in Paxinos and Franklin, 2008) were used for analysis (n = 3–12 sections per animal).Images of pE3-Aβ stained slides were taken at 4 × magnification (2.9 mm × 2.2 mm), including all stained striatal cells (Additional file 2: Figure S2A). To quantify neuronal numbers, parallel sections were stained with NeuN (Additional file 2: Figure S2C). To quantify only striatal cells a ROI was defined (Additional file 2: Figure S2D). All images were taken with constant exposure time and color balance with the Picture Frame Application 2.3 (Optronics) using a Nikon Eclipse 80i microscope (Nikon Instruments) with a MicroFIRE2.3A camera (Optronics), were saved as Tagged Image Files (TIF-24-bit) at a resolution of 213 dpi (1600 px × 1200 px) and analyzed with CP.

### Region of interest

To avoid neuronal density variances in the striatum due to high amount of neuropil in the medial striatum a rectangular ROI was defined in the basolateral striatum by using 10 × optical magnification (1.6 mm × 0.87 mm) with maximal lateral position with maximal possible distance of the lateral ventricle touching the external capsule (ec) and only including cells of the striatum (Additional file 2: Figure S2).

### Pipeline quantification of pE3-Aβ positive cells

Load images: Images at 4 × optical magnification (2.9 mm × 2.2 mm) including all pE3-Aβ stained cells of the striatum were loaded.Image math: Images were inverted. CP usually detects bright immunofluorescence signals with a dark background. By inverting immunohistochemical images, CP is able to distinguish dark objects from unstained background.Color to grey: Images were converted from RGB to greyscale. CP uses grayscale pictures.Identify primary objects: The minimum diameter was set to 1 pixel, and the maximum diameter was set to 10 pixels. Objects outside the diameter range were excluded. Cells were identified based on their immunohistochemical signal by the ‘robust background global’ algorithm. The threshold correction factor was set to 1.4. Threshold bounds were set from 0.0-1.0. Clumped objects were distinguished by staining intensity. Identified objects were called ‘pE3-Aβ positive cells’.Export to spreadsheet: After finishing the analysis, data was exported to *.xls for further statistical processing.

### Pipeline quantification of neurons in the basal lateral striatum

An example of the used pipeline is shown in Additional file 3: Figure S3A.Load images: Images at 10 × optical magnification (1.6 mm × 0.87 mm) of the ROI, including only NeuN stained cells of the striatum were loaded.Image math: Images were inverted.Color to grey: Images were converted from RGB to greyscale.Identify primary objects: The minimum diameter was set to 8 pixels, and the maximum diameter was set to 50 pixels. Objects outside the diameter range were excluded. Cells were identified based on their immunohistochemical signal by the ‘robust background global’ algorithm. The threshold correction factor was set to 1.1. Threshold bounds were set from 0.3-1.0. Clumped objects were distinguished by staining intensity. Identified objects were called ‘neurons’ (Additional file 3: Figure S3B).Export to spreadsheet: After finishing the analysis, data was exported to *.xls for further statistical processing.

## Competing interests

AB, SK, AA, WJ, CB, RS, and SG are or were employed by Ingenium Pharmaceuticals GmbH. HC and SS are or were employed by Probiodrug AG. HUD serves as Chief Scientific Officer of Probiodrug AG and as Managing Director of Ingenium Pharmaceuticals GmbH. SvH was consultant to the Probiodrug and Ingenium Group.

## Authors’ contributions

AB designed the study, made the experiments, developed the semi-automated image analysis, evaluated the data, and wrote the manuscript. SK and CB characterized the behavioral phenotype and evaluated the data. WJ evaluated the immunohistological methods. AA evaluated the immunohistological methods and wrote the manuscript. FC made the PPI experiments. HC designed the genetic construct. RS designed the genetic construct and made the genotyping experiments. SG and SS designed the study. HUD and SvH critically revised the manuscript. All authors read and approved the final manuscript.

## Supplementary Material

Additional file 1: Figure S1 Regional construct expression and processing. Example of coronal section stained with 6E10 of homozygous ETNA animals at the age of 3 months. Most severe immunoreactivity is found in the striatum (A). Also amygdala (B) showed strong Aβ expression and pE3-Aβ formation is observed at later time points. In hippocampus Aβ positive cells were observed in the pyramidal cell layer (C), but no pE3-Aβ could be observed up to the age of 9 months. In cortex (D) and brainstem (E) single positive cells were found. Click here for file

Additional file 2: Figure S2 Image examples and region of interest. Example of ETNA striatal coronal section stained with pE3-Aβ specific antibody, used to quantify pE3-Aβ positive cells by CP software (A). Images were taken at 4 x magnification and automatically inverted and transformed into grayscale (B). CP software detects numbers of stained cells and staining artifacts were excluded by low staining intensity and size above 10 pixels. To quantify neuronal numbers of ETNA striatal coronal sections were stained with NeuN-specific antibody. A ROI was defined as image at 10 x magnification (1.6 mm x 0.87 mm) in the basal, lateral striatum with maximal possible distance from lateral ventricle (LV), touching the external capsule (ec) and only including cells of the striatum (C). Example of ROI used for neuronal quantification (D). Click here for file

Additional file 3: Figure S3 Pipelines and image analysis. Pipeline (left) and ‘identify primary objects’ module (right) used to quantify neuronal numbers by CP software (A). Example of neuronal identification and segmentation of clumped cells (B). After inversion and transformation (right upper image), CP detects cells and segments clumped objects by staining intensity (green, right lower image). Click here for file
