# Supplementary figures and images for: Glutaminyl cyclase-mediated toxicity of pyroglutamate-beta amyloid induces striatal neurodegeneration
Source: BMC Neurosci. 2013 Oct 1;14:108. doi: 10.1186/1471-2202-14-108 (PMC3850634; doi:10.1186/1471-2202-14-108)

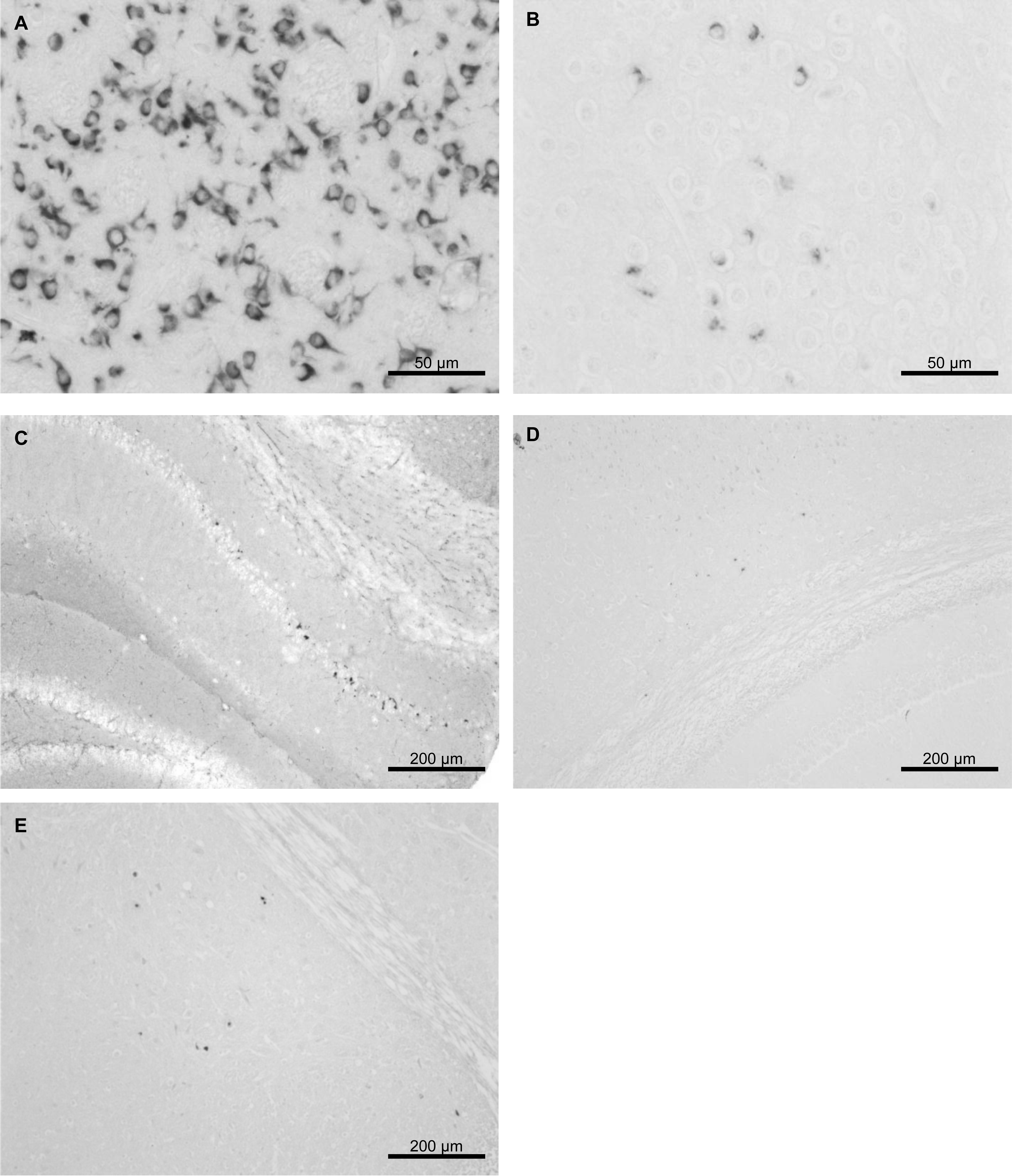

Supplement: Additional file 1: Figure S1 — Regional construct expression and processing. Example of coronal section stained with 6E10 of homozygous ETNA animals at the age of 3 months. Most severe immunoreactivity is found in the striatum (A). Also amygdala (B) showed strong Aβ expression and pE3-Aβ formation is observed at later time points. In hippocampus Aβ positive cells were observed in the pyramidal cell layer (C), but no pE3-Aβ could be observed up to the age of 9 months. In cortex (D) and brainstem (E) single positive cells were found. [file 1471-2202-14-108-S1.jpeg]

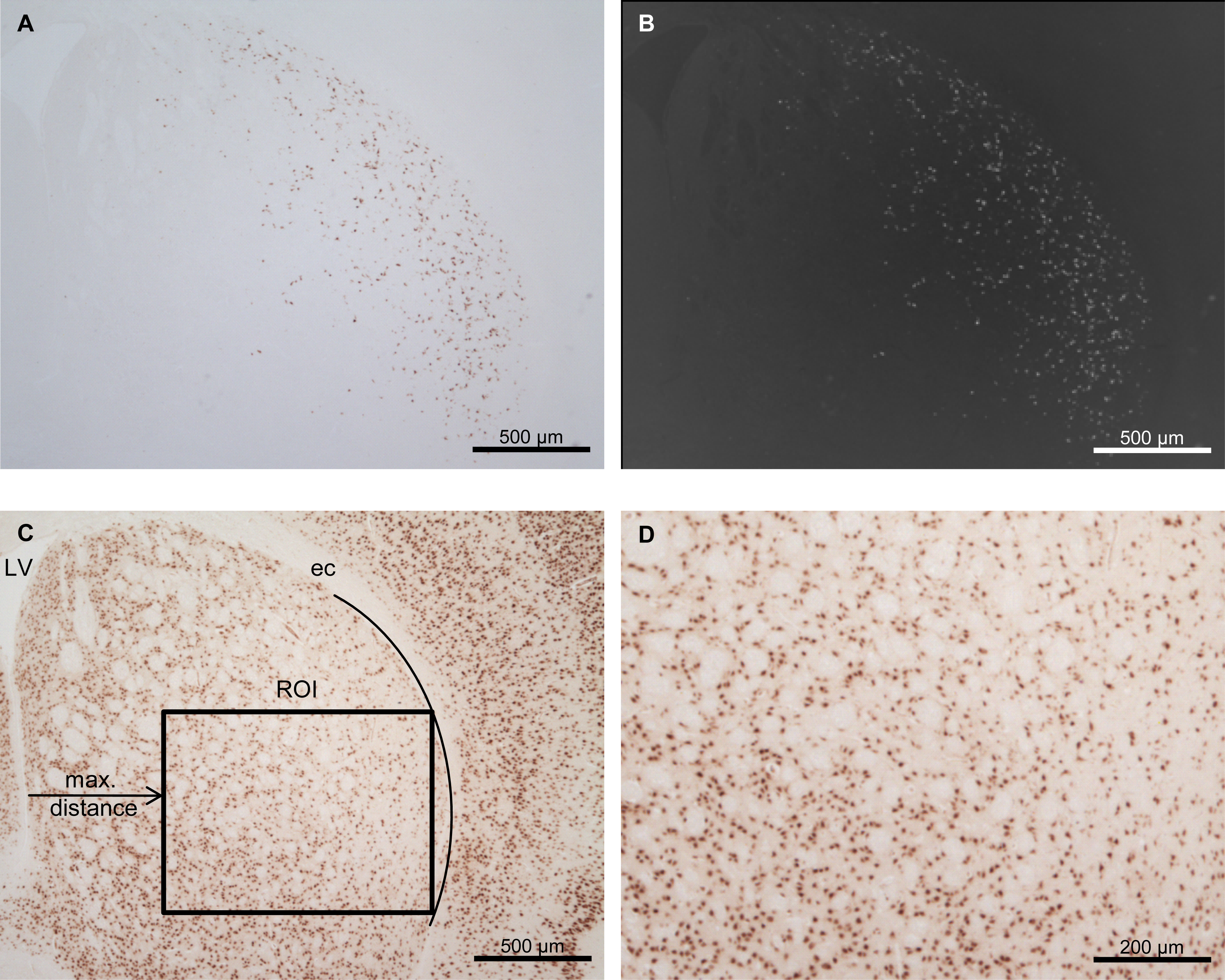

Supplement: Additional file 2: Figure S2 — Image examples and region of interest. Example of ETNA striatal coronal section stained with pE3-Aβ specific antibody, used to quantify pE3-Aβ positive cells by CP software (A). Images were taken at 4 x magnification and automatically inverted and transformed into grayscale (B). CP software detects numbers of stained cells and staining artifacts were excluded by low staining intensity and size above 10 pixels. To quantify neuronal numbers of ETNA striatal coronal sections were stained with NeuN-specific antibody. A ROI was defined as image at 10 x magnification (1.6 mm x 0.87 mm) in the basal, lateral striatum with maximal possible distance from lateral ventricle (LV), touching the external capsule (ec) and only including cells of the striatum (C). Example of ROI used for neuronal quantification (D). [file 1471-2202-14-108-S2.jpeg]

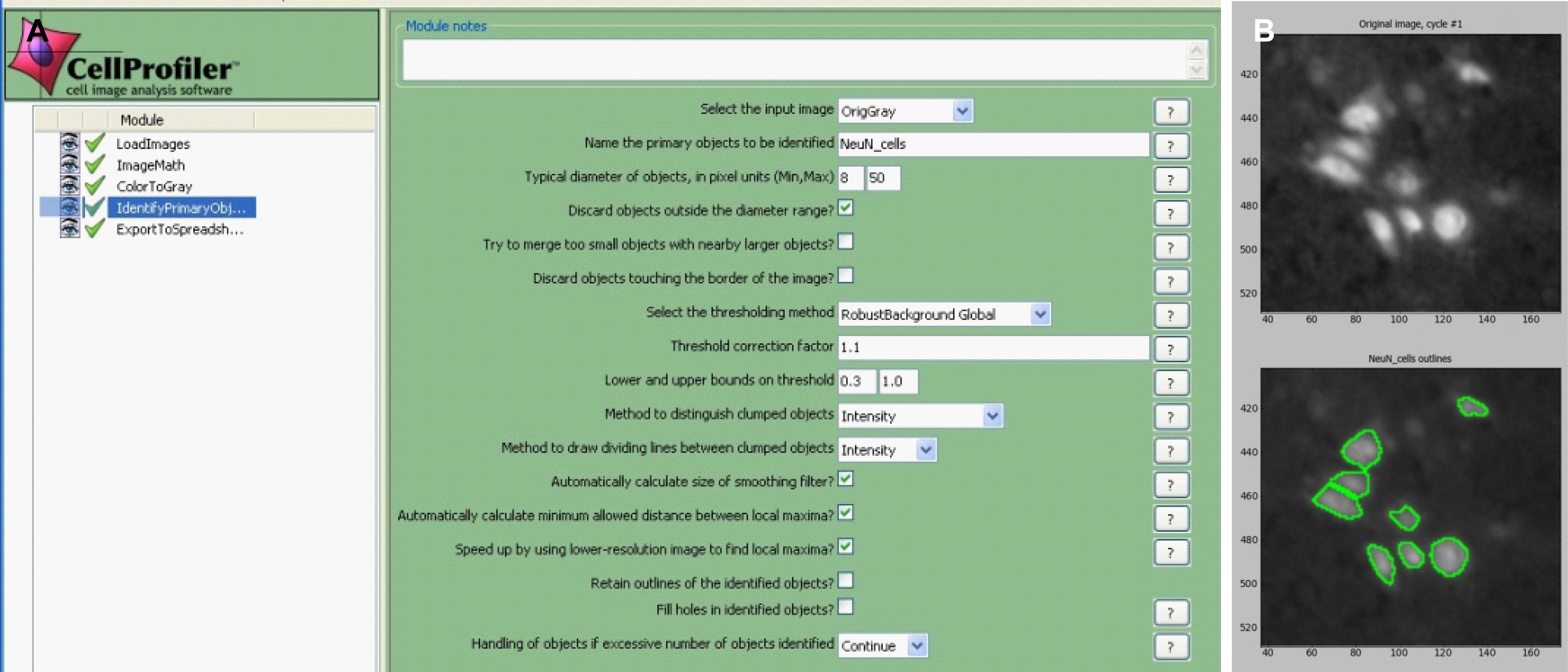

Supplement: Additional file 3: Figure S3 — Pipelines and image analysis. Pipeline (left) and ‘identify primary objects’ module (right) used to quantify neuronal numbers by CP software (A). Example of neuronal identification and segmentation of clumped cells (B). After inversion and transformation (right upper image), CP detects cells and segments clumped objects by staining intensity (green, right lower image). [file 1471-2202-14-108-S3.jpeg]
